# The Importance of Effective Population Size in Conservation and Biodiversity Monitoring

**DOI:** 10.1111/eva.70196

**Published:** 2026-01-17

**Authors:** Joachim Mergeay, Roberta Gargiulo, Yoshan Moodley, Isa‐Rita Russo

**Affiliations:** ^1^ Research Institute for Nature and Forest Geraardsbergen Belgium; ^2^ Royal Botanic Gardens, Kew Richmond UK; ^3^ University of Venda Thohoyandou South Africa; ^4^ Cardiff University Cardiff UK

## Abstract

Effective population size (*N*_e_) is a key concept in biology and conservation. Stripped to its bare essentials, it reflects how much genetic drift a population experiences, expressed as a number of individuals of an ideal theoretical population. Superficially, *N*_e_ seems like a fairly simple concept, but the more layers of the onion you peel, the more you feel like crying. Really understanding *N*_e_ in all its facets is daunting, as there are various temporal, spatial, biological, and mathematical ways in which *N*_e_ can be defined and approached, many of which are erroneously interchanged and often not distinguished. If that is not enough, understanding the intricacies and the assumptions of the many ways in which *N*_e_ can be calculated is required to make sense of the concept. This is why a special issue on this topic, especially in relation to biodiversity monitoring, is timely. We assembled 19 original papers, perspectives, and reviews on effective population size estimation in relation to conservation to help practitioners in conservation research and practical management see the forest for the trees with regards to *N*_e_.

## 1 Preface: A Tribute to Michael W. Bruford

This special issue is a celebration of international collaborations on conservation genetics, and a tribute to a great mind, friend, and advocate for conservation genetics, Michael W. Bruford. He left us way too early on April 13th, 2023, but his legacy in conservation biology, and especially in bridging the gap between conservation genetics, policy, and management, is strong and enduring (Hoban et al. 2023).

Mike pushed hard for the integration of genetics in global conservation policy through the Coalition for Conservation Genetics (CCG) and its member groups, which led to the inclusion of headline indicator A.4 (“the proportion of populations with *N*_e_ > 500”) in the Kunming‐Montréal Global Biodiversity Framework (KM GBF) of the Convention on Biological Diversity. This has cemented the concept of effective population size in international biodiversity policy and has finally brought genetic diversity into the realm of mainstream biodiversity monitoring. Many of the papers presented in this special issue have Mike's influence firmly etched in them.

## 2 A Short and Incomplete History of *N*_e_ and Its Calculation

In order to frame the different papers of this special issue, it is useful to browse through the history of *N*_e_, and see how the concept of *N*_e_ evolved over time, but also how principles have stood the test of time since the first paper on this topic appeared nearly 100 years ago. For a more detailed account and a more comprehensive taste of all the different types of *N*_e_ and how to calculate them, we refer to Wang (2016), Ryman et al. (2019), Nadachowska‐Brzyska et al. (2022), Delord et al. (2024) and Waples (2025).

The concept of an effective population size was first coined by Sewall Wright in 1931 as a means to measure the effect of genetic drift in a finite population. He deduced that a breeding population of size *N* increases inbreeding at a rate of 1/(2*N*) per generation (Wright 1922) and loses heterozygosity due to sampling variance at that same rate (Wright 1931). However, he noted that this number assumed an ideal population of *N* breeding individuals, which are produced each generation by random union of *N* male and *N* female gametes. He knew that in real populations this assumption was not likely met: “The number of surviving offspring left by different parents may vary tremendously either through selection or merely accidental causes, a condition which tends to reduce the effective *N* far below the actual number of parents or even of grandparents” (Wright 1931). He named this the effective population number, and understood that this concept was more important than the census size *N*_c_ (the number of potential parents) to understand how evolutionary processes affect patterns of genetic diversity.

By tracking the rate of increase of the inbreeding coefficient *F* through a pedigree, it became possible to calculate the effective size of a population (Wright and McPhee 1925). This way, Wright (1931) concluded that a horse breed studied by Calder (1928) of nearly 700 individuals had an effective size of only two dozen individuals, a staggering contrast. The reason for this was primarily a very skewed sex ratio due to the popularity of a few stallions.

More generally, the difference between the census and the effective size is determined by the variance in the number of progeny per parent (Crow and Morton 1955). If this reproductive variance (*V*_K_) is estimated together with the census size (*N*_c_), the effective size can be easily deduced, at least for monoecious diploid populations with discrete generations, as *N*_e_ = 4 (*N*_c_–2)/(*V*_k_ + 2). Modifications to accommodate other reproduction modes and overlapping generations were equally developed (Kimura and Crow 1963; Hill 1972, 1979). The sex ratio effective size formula (*N*_e_ = 4 *N*_m_ *N*_f_/(*N*_m_ + *N*_f_)) is actually a special case of this formula where the main source of variance is among sexes (Waples 2025).

In 1953 the structure of DNA was described (Watson and Crick 1953) and shortly thereafter the neutral theory of molecular evolution was developed (Kimura 1968). This theory described the expected gene diversity (*H*_e_) of a population at mutation‐drift equilibrium, as a function of *N*_e_ and the mutation rate. Therefore, under the assumptions of selective neutrality, mutation‐drift equilibrium and a known mutation rate, the (coalescent) *N*_e_ can be calculated from estimates of gene diversity.

Likewise, there is an equation describing the expected number of alleles (AR) as a function of *N*_e_ and sample size at mutation‐drift equilibrium (Ewens 1972). Since both *H*_e_ and AR are estimated from the same genetic data but react to demographic changes at different rates, they allow a test for deviations from mutation‐drift equilibrium, enabling the detection of past changes in *N*_e_ (Nei et al. 1975) under the assumption of neutrality. Flipping assumptions, we can also make inferences on selection at specific loci under the assumption of mutation‐drift equilibrium (Watterson 1977). Later on, this concept became very popular in the bottleneck test with microsatellite data (Cornuet and Luikart 1996). With the advent of actual DNA sequencing capabilities (Sanger and Coulson 1975), these principles to detect population size changes could also be applied to DNA sequences using nucleotide diversity and Watterson's theta as diversity and richness metrics, respectively (Tajima 1989). If the mutation rate was known, even the timing of these events and their respective past effective sizes could be estimated (Rogers and Harpending 1992).

Kingman developed coalescent theory using the fundamental assumption that the probability at which two allele copies in a particular generation share the same ancestor in the previous generation is 1/(2*N*_e_) (Kingman 1982). This allowed one to use DNA sequence information to estimate population genetic summary statistics like (the coalescent) *N*_e_, by tracing back how allele trees in an ideal population likely evolved through evolutionary processes. The coalescent *N*_e_ is the *N*_e_ that reflects the genetic diversity across the entire population's history up to the most recent common ancestor of all individuals of that population (Wakeley and Sargsyan 2009). Coalescent theory was later combined with analyses of the site frequency spectrum (Adams and Hudson 2004), which uses once more the deviation from mutation‐drift equilibrium by comparing a diversity metric (nucleotide diversity) and a richness metric (the number of segregating sites, Watterson's theta) across single nucleotide polymorphisms throughout the genome, to estimate changes in *N*_e_ and other demographic parameters across time (Gutenkunst et al. 2009; Wakeley 2021). Even a single diploid genome has enough information to allow the reconstruction of the ancient demographic history of a population (Li and Durbin 2011). Prior to the possibility of whole genome sequencing, the coalescent was also used to simulate specific demographic histories for more limited genotypic datasets (microsatellites or DNA sequences), and compare the outcome of each tested model with the actual empirical data to select the most likely scenario (Beaumont 1999; Drummond et al. 2005).

Since we know that gene diversity (*H*_e_) is lost at a rate of 1/(2*N*_e_) per generation in an ideal population, we can also estimate *N*_e_ when temporal samples are available. We can directly use the (average) reduction in *H*_e_, the variance in allele frequencies, or the increase in the inbreeding coefficient *F* over time to calculate *N*_e_, if we know the generation interval and can assume that mutations are negligible at that temporal scale (Nei and Tajima 1981; Waples 1989; Wang and Whitlock 2003).

William G. Hill described in 1981 a method to estimate *N*_e_ from linkage disequilibrium between alleles across pairs of neutrally evolving loci (Hill 1981), assuming neutrality and random mating. After small modifications to accommodate sample size bias (Waples 2006), this is to date the most popular method to estimate *N*_e_ (Waples and Do 2008). It also forms the basis for a generation‐by‐generation breakdown of changes in *N*_e_ across (recent) time from genome‐wide data of genetic variation, assuming the physical linkage among loci is known (Tenesa et al. 2007; Santiago et al. 2020).

Finally, genotypic data allow one to calculate the frequency of siblings in a sample. This is a direct function of the effective number of parents in the previous generation, thus providing a sibship‐based method for estimating contemporary *N*_e_ (Wang 2009).

As a bonus, there is a method using approximate Bayesian computation that combines several of the above population genetic summary statistics (LD, *H*_e_, AR, …) to estimate the contemporary local *N*_e_, given a set of priors (Tallmon et al. 2008). In summary, there are several families of methods to estimate *N*_e_. Table 1 shows the different papers in this special issue and how they relate to each method.

**TABLE 1:** Overview of the six classes of methods to estimate *N*_e_ or detect demographic changes, and the different papers in this special issue using them. Delord et al. (2024) and Fedorca et al. (2024) discuss these principles and methods in more detail.

Principle	Elaboration	Main references	Used in this SI by
Demographic (includes sex ratio method). Estimates contemporary *N*_e_	The variance in reproductive success is inversely proportioned to the *N*_e_. The sex ratio method is a special case where the only source of non‐ideal variance is among sexes.	Wright (1931), Kimura and Crow (1963), Hill (1972, 1979)	Allendorf et al. (2024), Delord et al. (2024), Fedorca et al. (2024), Kvalnes et al. (2024), Mergeay (2024), Mergeay et al. (2024), Waples (2024)
*H*_e_ and AR at mutation‐drift equilibrium Includes tests for demographic declines and expansion, and the timing of events	At mutation‐drift equilibrium *N*_e_ predicts both *H*_e_ and AR. *H*_e_ can then be used to calculate (coalescent) *N*_e_. Since *H*_e_ and AR are estimated from the same sample we can test for deviations of mutation‐drift equilibrium and identify the direction, as well as the timing of major past demographic events	Kimura (1968), Ewens (1972), Nei et al. (1975), Tajima (1989), Rogers and Harpending (1992), Cornuet and Luikart (1996)	Allendorf et al. (2024), Clark et al. (2024), da Silva et al. (2025), Delord et al. (2025), Fedorca et al. (2024), Mergeay (2024), Parreira et al. (2025), Thomas et al. (2025)
Coalescent theory	Coalescent theory models how allele lineages merge going backward in time (gene genealogies) as a function of the mutation rate, *N*_e_ and gene flow. Derived methods estimate the demographic parameters that best explain the genetic data	Kingman (1982), Beaumont (1999), Drummond et al. (2005), Gutenkunst et al. (2009), Wakeley (2021)	da Silva et al. (2025), Delord et al. (2024), Delord et al. (2025), Fedorca et al. (2024), Parreira et al. (2025)
Temporal methods	The change in inbreeding or the amount of drift that occurred across two times points predicts *N*_e_. Also the variance in allele frequencies across time points scales inversely with *N*_e_	Wright and McPhee (1925), Nei and Tajima (1981), Waples (1989)	Fedorca et al. (2024), Lévêque et al. (2024)
Linkage disequilibrium (LD)	Estimates *N*_e_ from nonrandom associations between alleles at different loci: small *N*_e_ increases LD due to genetic drift. Contemporary *N*_e_ is estimated from physically unlinked loci, while past *N*_e_ can be estimated from the decay of physical linkage between mapped loci	Hill (1981), Waples and Do (2008), Tenesa et al. (2007), Santiago et al. (2020)	Bertram et al. (2024), Cox et al. (2024), da Silva et al. (2025), Delord et al. (2025), Fedorca et al. (2024), Gargiulo et al. (2024), Lévêque et al. (2024), Mergeay et al. (2024), Pavlova et al. (2024), Pérez‐Sorribes et al. (2024), Robinson et al. (2024), Thomas et al. (2025)
Sibship	Estimates *N*_e_ by identifying full‐ and half‐sibling relationships among sampled individuals, with the frequency of sibships being inversely proportioned to the number of parents	Wang (2009)	Cox et al. (2024), Fedorca et al. (2024), Lévêque et al. (2024), Mergeay et al. (2024), Pavlova et al. (2024), Robinson et al. (2024)

It should be noted that there is a very diverse nomenclature surrounding the concept of *N*_e_, with lots of “flavours” (Waples 2022) depending on the biomathematical context (inbreeding, variance, linkage disequilibrium, gene diversity, eigenvalue, additive variance *N*_e_), the spatial scale (Ryman et al. 2019), and the temporal continuum (Nadachowska‐Brzyska et al. 2022). All too often, they are just called “effective population size” without modifiers of what was actually estimated and what they represent. This can cause a Babylonian speech confusion in which even conservation geneticists get tangled up and confused. Focusing on the contemporary effective size is particularly useful in conservation, as it provides a future perspective of loss of genetic diversity, rather than looking in hindsight at the population histories that shaped the contemporary levels of genetic variation.

All of the methods that are used to estimate *N*_e_ go with a manual that should not be ignored (Waples 2025). But even with a manual, our understanding of the sensitivity of each of these methods to its (explicit or implicit) assumptions is incomplete but growing.

## 3 Effective Population Size in International Biodiversity Policy

Since the inception of the Convention on Biological Diversity in 1993, genetic diversity has been acknowledged as one of three pillars of biodiversity, and countries were asked to monitor and track genetic diversity issues in their reports every 5 years. In practice, genetic diversity has been very poorly covered in these reports, especially in wild species (Hoban et al. 2021). In 2022, however, the CBD adopted headline indicator A.4 through the Kunming‐Montréal Global Biodiversity Framework, which directly links to genetic diversity through the concept of an effective population size. This so‐called Ne500 indicator represents the proportion of populations per species with an effective size larger than 500. Above this effective size, isolated populations are generally considered large enough to maintain evolutionary potential in perpetuity (Jamieson and Allendorf 2012; Hoban et al. 2020). In practice, few assemblages of individuals we tend to call populations are really isolated, so this criterion should really be applied to metapopulations (Waples 2024), defined as sets of subpopulations internally connected by at least one effective migrant per generation.

This milestone in conservation genetics puts a lot of responsibility on our shoulders: estimating *N*_e_ is not an easy task and has a lot of pitfalls (Waples 2025). This means we need to improve our knowledge on how to apply which method under what biological circumstances and adapt our methodological approaches to the question we're trying to answer, instead of using default popgen sampling strategies and trying to fit post hoc one or more *N*_e_ estimation methods and hope for the best. Several papers in this special issue highlight how critical sample size and sampling design are to estimating *N*_e_ confidently (Bertram et al. 2024; Cox et al. 2024; Mergeay et al. 2024; Parreira et al. 2025).

### 3.1 Using Proxies to Estimate *N*_e_

In the guidelines for indicator A.4 of the KM GBF, countries are advised to report on at least 100 species, often across multiple populations per species. In practice, it is unfeasible to estimate for hundreds of populations *N*_e_ with molecular tools or from life history tables. There are, however, shortcuts that are often good enough to give an overall impression of the *N*_e_ of a population: if we know the ratio of the effective size *N*_e_ to the census size *N*_c_, we can deduce the expected *N*_e_ from population counts, for which data are often readily available through other forms of biological monitoring (Mastretta‐Yanes, da Silva, et al. 2024). In the absence of population counts, the area of occupation of a population may be used if we can assume a specific density of individuals. This cascade of assumptions may not yield accurate estimates of *N*_e_, but since the headline indicator A.4 only requires knowing if the *N*_e_ is below or above the 500 threshold, this can often be good enough (Hoban et al. 2024; Mastretta‐Yanes, Suárez, et al. 2024).

## 4 Advances From Contributions to This Special Issue

This special issue clearly has a single central theme, but we have three main tracks on which research was focused across the 19 papers, discussed below.

### 4.1 Understanding *N*_e_, *N*_b_, *N*_c_ and Their Relations

We have four papers that take a deep dive into *N*_e_ and other aspects of population size that influence the evolutionary trajectories of populations.

Who other than Robin S. Waples to review the theory behind the drivers of the *N*_e_/*N*_c_ ratio (Waples 2024), which often boils down to a single variable: the variance in lifetime reproductive success in a population. If this variance is large, the effective size is small relative to *N*_c_. What causes the variance to be large or small is important to know, and this is clearly dissected and partitioned into different components.

Delord et al. (2024) provide an excellent review of the *N*_e_/*N*_c_ ratio, the challenges in estimating either in marine fish populations and how to relate them to each other, and further discuss the drivers of the *N*_e_/*N*_c_ ratio in marine fish populations.

Allendorf et al. (2024) point out that allelic richness (aka allelic variation) is a key indicator of long‐term adaptive changes in a population and can be effectively monitored using its linear relationship with census size. They propose that monitoring census size alongside effective population size can better capture both immediate genetic changes and long‐term adaptive changes and advocate for conservation strategies that consider both *N*_e_ and allelic variation. This way, they argue the CBD should include additional metrics for genetic conservation beyond the Ne500 criterion.

Along similar lines, Mergeay (2024) argues that both *N*_e_ and *N*_c_ are essential for properly understanding evolutionary dynamics. He uses information theory to show that we can consider *N*_c_ as the population size richness and *N*_e_ as the population size diversity, just like we also have gene diversity (and the derived effective number of alleles) and allelic richness as complementary summary statistics in population genetics. This highlights how *N*_e_ and *N*_c_ are two faces of the same coin, with different impacts on evolutionary trajectories.

### 4.2 Testing and Tailoring *N*_e_ Estimation Methodology

Next, we have a series of papers focused on testing how well *N*_e_ estimation methods perform when underlying model assumptions are violated, and on tailoring sampling designs to life history traits. Mixed with this are underlying conservation and management questions.

There is a vast array of methods to estimate *N*_e_, each with different sets of assumptions, and we often lack the tools or the possibilities to test these assumptions for particular settings. Moreover, these assumptions are very rarely met, and many methods support some violations. But in truth, we often ignore how sensitive these methods really are to violations of the sometimes silent assumptions, and it has become rather standard (yet sometimes questionable) practice to try out different methods and pick the numbers ‘that make sense’. Furthermore, there is no single summary statistic that is “THE” *N*_e_, unless you are dealing with a population whose entire history happened in isolation and is at mutation‐drift equilibrium.

Delord et al. (2025) focus on pelagic fish populations and review theory on *N*_e_ in that context. They provide a simulation framework to test how well LD‐based and coalescent‐based methods (including those using site frequency spectra) work for genomic datasets. Marine fish populations' effective size has long been underestimated, for a variety of reasons discussed in the paper. This helps researchers assess method reliability, especially for large, complex populations.

Fedorca et al. (2024) report on a workshop from COST Action G‐BiKE and address challenges in estimating *N*_e_ for conservation. They emphasize that *N*_e_ estimation methods rely on simplifying assumptions (e.g., no immigration, panmixia, equilibrium), which are often violated in real, fragmented populations, potentially biasing results. The workshop aimed to test method sensitivities under realistic scenarios, propose improved analytical strategies, and bridge the gap between theoretical knowledge and practical conservation applications. It is clear that GONe (Santiago et al. 2020) has become a game changer in conservation genetics, but its sensitivity to model assumptions still needs further testing. Two studies were initiated from this G‐BiKE workshop to test the efficacy of GONe under varying conditions. GONe exploits linkage information among loci to estimate *N*_e_ at different points in time: unlinked or loosely linked loci provide information about *N*_e_ in recent generations, while physically linked loci inform *N*_e_ estimates in the past (up to 200 generations ago). This method is innovative in that it can provide “recent historical” *N*_e_ estimates leveraging genomic datasets, provided that (i) the assumptions of the linkage disequilibrium method are met and (ii) a sufficient number of loci (and SNPs) mapped to chromosomes are available. Gargiulo et al. (2024) focus on how GONe deals with imperfect datasets in plant species (missing data, small to large numbers of SNPs, and lack of a complete reference genome or linkage map). Plants often have complex life histories (large, continuous ranges, overlapping generations, or unusual reproductive systems), which can lead to inaccurate and biased *N*_e_ estimates. Pérez‐Sorribes et al. (2024) took advantage of well curated genomic datasets from two wolf populations with known histories to verify how well GONe reconstructs the known population histories.

Continuing with wolves, Mergeay et al. (2024) use a database of wolf life history traits to accurately estimate the contemporary *N*_e_ of the German wolf population from the variance in reproductive success. Using this as a reference, they compare the performance of a sibship and a LD‐based method, and contrast varying sensitivities of these methods to different spatial sampling designs across different wolf populations. Incidentally, they show the number of packs is a really good approximation of the effective size, which is very relevant for conservation and monitoring.

Along similar lines, Cox et al. (2024) explore the sensitivity of two *N*_e_ estimation methods to different spatial sampling designs in an exhaustively sampled population of moor frogs, and show that even subtle spatial genetic structure strongly impacts Ne estimates. Their results corroborate that sampling schemes typical for classical population genetic studies are not always good enough for *N*_e_ estimation.

Bertram et al. (2024) focus on the influence of sample size to estimate *N*_b_ and *N*_e_ for heavily exploited marine fish populations of the Australasian snapper, and the question of whether to use single age cohorts versus mixed age samples.

It has been clear for a while that we need to account for spatial genetic structure when dealing with *N*_e_ estimations, expansions and bottlenecks (Chikhi et al. 2010). Parreira et al. (2025) expand on this by showing that social genetic structure (caused by living in social groups) can have an equally strong influence on estimations of past population changes.

Finally, Clark et al. (2024) explore through simulations how using age‐structured genetic data can improve detection of recent population declines in long‐lived species, such as trees, turtles, or some fishes. They show that sometimes it is better to treat the genomes of older individuals as pseudo‐temporal sampling compared with those of younger individuals.

### 4.3 Conservation and Management Informed by *N*_e_

The third section of papers provides case studies where *N*_e_ estimates are mainly used to provide conservation and management advice for particular species and populations, but which often still have a component of “testing and tailoring”.

Lévêque et al. (2024) try to estimate Ne across a set of peri‐urban metapopulations of the southern damselfly, using SNPs and microsatellites for single populations, metapopulations, and with a variety of approaches. While they conclude the metapopulation *N*_e_ values are likely large enough to maintain evolutionary potential, they highlight the difficulty in estimating *N*_e_ reliably for subpopulations, pointing at violations of model assumptions.

Kvalnes et al. (2024) use a life history based approach to calculate the effective size of the Norwegian reindeer population, and next simulate the effect of different harvest and disease management regimes on the effective size and overall conservation outlook of the population.

Robinson et al. (2024) show that the effective number of brook trout breeders (*N*_b_) is a good indicator of population status, showing clear links between Nb and a set of environmental drivers of population change.

Pavlova et al. (2024) study the Macquarie perch in Australia in a genetically fragmented riverscape setting and used a variety of techniques and analyses to provide concrete management recommendations needed to avoid extinction.

Thomas et al. (2025) tackle the population recovery of Eurasian otters in the United Kingdom from the perspective of effective population size. They show that, in spite of ongoing demographic recovery, the population is still short of reaching the Ne500 target for long‐term sustainability.

Da Silva et al. (2025) infer the recent and ancient demographic history of Western Chimpanzee populations using a wide range of *N*_e_ estimation methods. This helps to understand the evolutionary history and current demography of this critically endangered great ape, and should help to inform management and conservation decisions.

## 5 What's Next in *N*_e_ Research for Conservation?

### 5.1 Guidelines and Guidance

The plethora of methods to estimate *N*_e_ and their underlying assumptions, conditions, and sampling requirements are daunting for anyone. If anything, practitioners need a set of guidelines on how to estimate *N*_e_, tailored to their conditions. There is, however, no silver bullet: like many of the papers in this special issue demonstrate, there is no single best methodology that is applicable across all populations, not even for a particular species. For those getting started, heed the warning of Waples (2025): read the manual! Study your species, learn its intricacies, and reach out for advice; there is a large conservation genetic community of experts who are knowledgeable and who want to promote the uptake of genetic methods in conservation. The Coalition for Conservation Genetics (Kershaw et al. 2022) has a handful of partner organizations with dozens to hundreds of members each. And take advantage of the papers in this special issue to study the quips and quirks of *N*_e_.

### 5.2 Reliable *N*_e_/*N*_c_ Ratios

The methodological choices to estimate *N*_e_ also depend on the precision required: for Headline Indicator A.4 for the CBD, for example, it suffices to evaluate whether or not the effective size is (well above or below) 500. Often, census size estimates or other derived proxies (such as the available habitat area for species with little variation in the density of habitat use) can be enough if the *N*_e_/*N*_c_ ratio applied for that species is sufficiently robust (Mastretta‐Yanes, Suárez, et al. 2024).

The typically applied default *N*_e_/*N*_c_ ratio of 10% is often conservative and may vary strongly across species, mostly as a function of life history traits. There is absolutely a tremendous need to improve our general understanding of *N*_e_/*N*_c_ ratios across the tree of life if we are to report on genetic indicators. For example, it has long been thought that marine teleosts have tiny *N*_e_/*N*_c_ ratios, based on calculated *N*_e_ values and estimated fish stock sizes (see for example Bertram et al. 2024), but it is often still unclear if these are reliable or result from the violation of model assumptions when estimating *N*_e_ (Marandel et al. 2019). Even for easier species, published *N*_e_/*N*_c_ ratios sometimes vary 20fold (see supporting information in Hoban et al. 2020), and it is unlikely that these differences always reflect biological differences across the studied populations.

Improving our knowledge on the *N*_e_/*N*_c_ ratio means having robust and reliable *N*_e_ and *N*_c_ estimates, estimated at the same spatial and temporal scales (Palstra and Fraser 2012; Delord et al. 2024; Waples 2024), and with high precision. Currently, the published literature on this is still scarce, with only a few hundred species covered and often large uncertainties (Hoban et al. 2020). Since we know the *N*_e_/*N*_c_ ratio is to a large extent influenced by a few life history traits (Waples et al. 2013), there is a path towards predicting the *N*_e_/*N*_c_ ratio from robust *N*_e_ and *N*_c_ inferences and life history trait information across the tree of life.

## 6 Conclusions

Estimating *N*_e_ is important, but also fraught with difficulties. We hope the papers in this special issue will help the reader to improve their understanding of *N*_e_ in conservation and to help advance the uptake of genetic diversity in policy and management.

## Funding

We thank Cost Actions 18134 G‐BiKE and CA23121 GENOA for financial support to organize meetings on the topics covered in this paper. JM was supported by Biodiversa+ project GINAMO.

## Conflicts of Interest

The authors are Editorial Board members of Evolutionary Applications of this article. To minimize bias, they were excluded from all editorial decision‐making related to the acceptance of this article for publication.

## Data Availability

Data sharing not applicable to this article as no datasets were generated or analysed during the current study.
